# Sequencing quality assessment tools to enable data-driven informatics for high throughput genomics

**DOI:** 10.3389/fgene.2013.00288

**Published:** 2013-12-17

**Authors:** Richard M. Leggett, Ricardo H. Ramirez-Gonzalez, Bernardo J. Clavijo, Darren Waite, Robert P. Davey

**Affiliations:** The Genome Analysis CentreNorwich, UK

**Keywords:** quality control, sequence analysis, QC, NGS data analysis, bioinformatics tools, run statistics, quality assessment and improvement, contamination screening

## Abstract

The processes of quality assessment and control are an active area of research at The Genome Analysis Centre (TGAC). Unlike other sequencing centers that often concentrate on a certain species or technology, TGAC applies expertise in genomics and bioinformatics to a wide range of projects, often requiring bespoke wet lab and *in silico* workflows. TGAC is fortunate to have access to a diverse range of sequencing and analysis platforms, and we are at the forefront of investigations into library quality and sequence data assessment. We have developed and implemented a number of algorithms, tools, pipelines and packages to ascertain, store, and expose quality metrics across a number of next-generation sequencing platforms, allowing rapid and in-depth cross-platform Quality Control (QC) bioinformatics. In this review, we describe these tools as a vehicle for data-driven informatics, offering the potential to provide richer context for downstream analysis and to inform experimental design.

## 1. Introduction

The Genome Analysis Centre (TGAC) is a UK institute founded in 2009, strategically supported by the Biotechnology and Biological Sciences Research Council (BBSRC). The institute predominantly carries out research programmes in genomics and bioinformatics, collaborating with groups around the world, but also provides customer sequencing and bioinformatics services, in particular to other BBSRC grant holders.

TGAC hosts Illumina MiSeq and HiSeq, Pacific Biosciences RS, Life Technologies Ion Proton and Roche 454 machines, as well as complimentary technologies such as the OpGen Argus optical mapper. The PacBio and OpGen are starting to be used more regularly and for a wider array of applications, but our workhorses remain the MiSeq and HiSeq.

The applications of sequencing data at TGAC are many and varied. Assembly work is a core focus, encompassing a diverse range of organisms sequenced from simple microbes to complex crop genomes with high frequency of repeat structures and ploidy number. We also undertake variant analysis, target exome capture, metagenomic analysis, RNA-Seq (transcriptome sequencing) and RAD-Seq (Restriction site Associated DNA sequencing). Whatever the application, we believe that one of the most critical ways to achieve high quality results is to *know your data*. This includes not only understanding the lab protocols and sequencing technologies, but also understanding the data that emerges from the machines, in terms of quality, contamination and content. Interrogation of data is not always contextualized by a classical hypothesis-driven scenario. An experiment can be initially well-designed for a given biological hypothesis and the data produced can seem sufficient at first glance, but subsequently deemed suboptimal following analysis and bioinformatic investigation. Knowledge gained from in-depth informatics can feed back into the experimental design with no prior context necessary—indeed, assumed biases applied at this level can mask real data problems. This is *data-driven informatics*, in which *knowing the data* itself informs downstream analysis and future experimental design. For example, the kmer content and heterozygosity of a dataset (see section 3.1) can dictate appropriate assembly approaches, the potential need for further sequencing, or ultimately changes in hypothesis formation. Similarly, the application of the NextClip tool (see section 3.2) which, through organism-neutral analysis of the underlying read data produced from the stock Nextera Long Mate Pair (LMP) protocol, has enabled the library preparation protocols to be improved resulting in tighter insert size distributions and leading to improved assemblies.

In this paper, we describe our Primary Analysis Pipeline (PAP), as well as a number of related tools and techniques that we apply to initial analysis of sequencing data. Where possible, we have sought to design or adopt software tools that can be used cross-platform, as this makes it easier to integrate data from multiple platforms and to design pipelines as new platforms emerge. Where software is developed in house, the institute policy is to release the source code with an open source licence and in so doing, we hope to be able to contribute to the wider bioinformatics community.

## 2. Primary analysis pipeline

**Figure 1:**
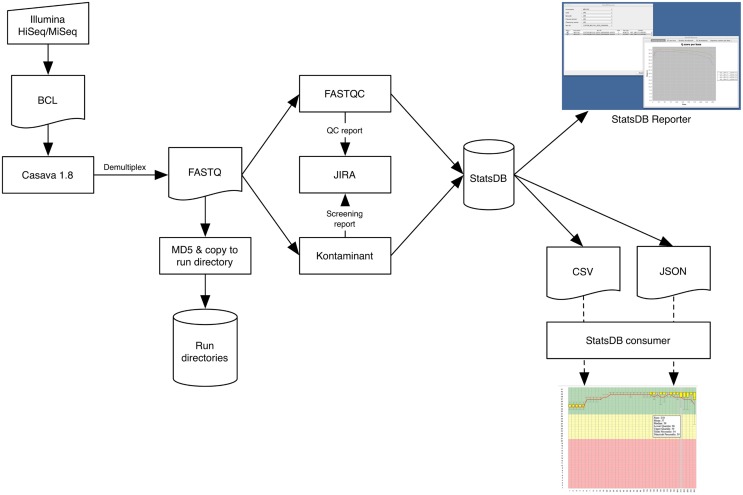
**Data flow through the Primary Analysis Pipeline, focused on the Illumina platform**.

The main stages of our PAP are summarized in Figure 1. The pipeline is designed to be initiated from within TGAC's MISO Laboratory Information Management System (LIMS) (Davey et al., 2013), but can also be manually executed. Once the run has completed on the instrument, an operator can use MISO's web interfaces to start the PAP and to monitor its progress. The Sequencing Operations team use the Atlassian JIRA issue tracking system (Atlassian, 2013) to review sequencing run analysis and to note anything abnormal about a particular sample or run, so the PAP will also automatically write Quality Control (QC) information to the associated run ticket. This information is then available for project managers or bioinformaticians waiting for the data. Accurate timely notification of issues is essential for scaling up any pipeline, so having a smooth robust workflow in place that is able to highlight potential bottlenecks is always a key development focus.

### 2.1. Demultiplexing and format conversion

Multiplex sequencing is a common technique to increase sample throughput and reduce cost. Such runs need to be processed to deconvolute each sample into a respective data file. For the PacBio and Proton platforms, demultiplexing is effectively carried out on instrument, whereas for Illumina, we undertake this on our High Performance Computing (HPC) cluster. In the latter case, the PAP initially calls Illumina's CASAVA software to demultiplex samples and convert BCL files to FASTQ format files. MISO automatically produces required sample sheets and defines the bases mask, elucidated from the run metadata, which tells CASAVA whether cycles are part of reads or indexes.

### 2.2. Metric generation

The PAP utilizes FastQC (Andrews, 2010) for quality analysis of FASTQ files, which is able to report a wide range of information related to the quality profile of the reads. Additionally, FastQC will assess GC content, over-abundance of adaptors and over-represented sequence, from which an indication of PCR duplication rate may be inferred. The PAP extracts certain key information, particularly the number of reads and average length of read with quality score at 30 or above, and produces a per-sample summary on the JIRA ticket as well as a PDF file containing all the main FastQC plots.

Project managers examine these summary reports, identifying potential areas of further investigation either experimentally or computationally, liasing with collaborators or bioinformaticians, respectively. The summary will highlight in red any samples for which the quality or yield is lower than an expected minimum. Of particular note are the base-by-base quality plots, which can quickly highlight issues with a run and enable project managers to request further processing (such as trimming) or plan for rescheduling of runs. Low yields for particular barcodes can indicate library preparation or index specification problems which may necessitate re-running of demultiplexing. Reporting on the frequency of undetermined indices is standard across all runs allowing any barcoding issues to be detected. The graph of mean GC percentage against number of reads allows easy identification of potential contamination, which typically appears in the graph as deviation from a normal distribution or as a second peak. Such observations are subsequently confirmed by other tools, such as Kontaminant, described below.

### 2.3. Contamination screening

Contamination screening is based upon TGAC's own screening and filtering tool, Kontaminant (Ramirez-Gonzalez, 2013), which utilizes a kmer-based approach. Kmers are formed by moving a window of size *k* nucleotides, base-by-base over a read. At each window position, a kmer is generated that will overlap with the next kmer in all but one base. For Kontaminant, we typically use a kmer size of 21 and for reasons of speed, we subsample 10% of each read file. Kmers from a read are compared against hash tables which are pre-generated from whole references or single chromosomes of larger genome organisms, e.g., human. Kontaminant will report the percentage of reads containing one or more contaminant kmers, as well as the percentage of the contaminant genome that is covered by the entire read file. These two figures together provide a reliable indication of true contamination. For example, screening a bacterial sample will often turn up a significant percentage of reads containing one or more *E. coli* kmers; however, the percentage of the *E. coli* reference covered is typically low, indicating that these are simply shared kmers between bacterial species rather than true contamination.

Typical contaminant references comprise Phi X, *E. coli*, human chromosome 17, plant chloroplasts, and BAC vectors. For projects where there is high risk of a particular contaminant, additional screening references will be utilized. Similarly, if downstream analysis, such as assembly, provides evidence of contamination not routinely screened for, additional screening can be carried out against datasets generated by the same instrument or with the same reagent kits.

A report of contamination levels for each sample is appended to the JIRA ticket for the run. As routine filtering of reads runs the risk of losing important data, filtering is requested based on assessment of true contamination levels and whether it is preferable to filter the reads initially or to extract contaminant contigs after assembly of the reads.

### 2.4. QC analysis storage

Output from metric generation and contamination screening analyses represent informative statistics concerning the overall quality of the run. These are stored using another TGAC tool, StatsDB (Ramirez-Gonzalez et al., 2013), which provides a flexible database and API for storing any run metric or metadata. For the PAP, StatsDB is used to store metadata about the sequencer software and chemistry versions used, as well as complete FastQC data and contamination screening output.

The core of StatsDB is a flexible MySQL schema that allows attributes to be defined and stored on a per-run, per-base, or per-range (for example, bases 1–5) basis. Perl and Java APIs allow data to be easily written to and read from the database with minimal knowledge of the underlying structure. We envisage, and have implemented instances of, two types of program communicating with the StatsDB API—parsers and consumers. Parsers are simple scripts that process and store the output of tools such as FastQC. Consumer tools query StatsDB and present data to a user.

Understanding how sequencing performance varies across lane, instrument and time is not easily achieved with current QC tools which typically focus on a single sample, lane or run. Parsing the output of these tools into StatsDB brings the data into a shared context that enables cross-sequencer run metrics and historical data to be directly comparable, represented as consolidated tables and graphs. Consumer implementations such as MISO and the StatsDB Reporter tool provide easy access to these powerful multi-faceted analyses.

### 2.5. Data exposure

The final stage of the core PAP is to MD5 checksum the processed reads and copy them from a run directory into a common storage staging area, accessible as read-only by the whole institute and organized by project and run alias. This enables easy access to the data by bioinformaticians for downstream processing, by project managers who will pass it on to collaborators and customers, or by other internal data management systems, such as the iRODS (iRODS, 2013) data grid system. Providing the iRODS layer over our file system, we can annotate our data with descriptive metadata which enables consolidated searching and discovery of grouped datasets, e.g., by an overarching project alias, or a more specific query such as “all RAD-Seq data produced last month from HiSeq 2500”. With data management and analysis frameworks such as the iPlant Collaborative (iPlant, 2013) also implementing the iRODS stack, and potential integration with Galaxy (Goecks et al., 2010) via the iRODS APIs, it becomes possible to automatically and rapidly expose sequence data for downstream analysis as a matter of course.

### 2.6. Scripting

The PAP is implemented as a series of Perl and shell scripts that execute the individual software components. MISO initiates each step of the PAP using Conan (Burdett, 2013), a Java library that provides a lightweight but flexible developer-orientated framework for pipeline implementation. Jobs and dependencies are managed using the Platform LSF scheduling system on the TGAC HPC cluster. Eight 16-core 48GB RAM cluster nodes are reserved for the PAP, restricting the software that runs on them and ensuring the PAP always has priority scheduling.

Due to the bespoke nature of the scripts, tailored to institute-specific infrastructure, they are not immediately useful for release, but we hope to make as much of the PAP as open as possible in the future.

## 3. Supplementary pipelines

There are a number of additional pipelines that can be executed manually, depending on the eventual application of the read data.

### 3.1. Kmer space metrics

It is often the case that the raw sequencing data we generate will go on to be assembled. Most assemblers for next generation sequence data are based on the concept of de Bruijn graphs, where nodes are formed by kmers and edges represent single base differences between the kmers. Contigs are formed by walking through the graph, building contiguous sequence from the kmers and the edges. Before attempting assembly, kmer counting and the resulting interpretation can provide revealing insights into data quality and the attributes of the organism.

We perform kmer assessment using TGAC's own Kmer Analysis Toolkit (KAT) (Clavijo et al., 2013). KAT analyses hash files created with Jellyfish (Marcais and Kingsford, 2011), typically using a *k* value of 31. The hashes are generated during the PAP run and are retained for further use, as KAT is employed for downstream analysis for assembly projects. There are two distinct outputs from the kmer analysis: a simple spectra plot for read 1, read 2, and their sum; and a shared and unique content comparison.

**Figure 2:**
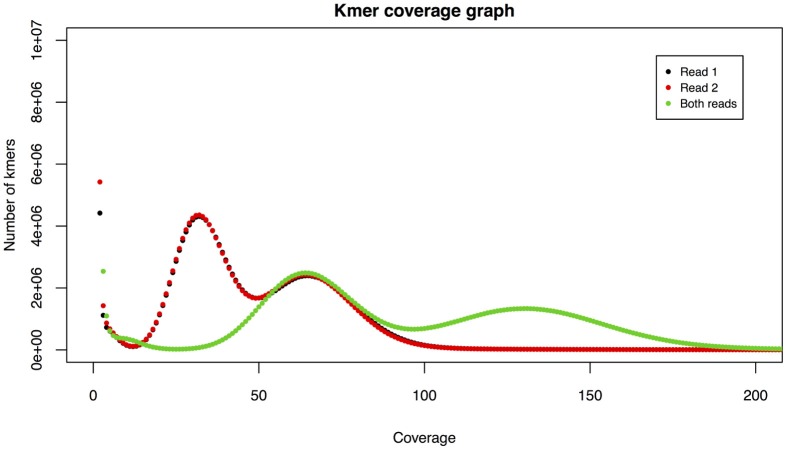
**Example kmer spectra**. An initial peak around coverage 1 is indicative of sequencing errors. Two further peaks indicate heterozygosity.

The kmer spectra plot shows coverage against count of kmers with a given coverage. We make a separate count for read 1, read 2, and then for both reads and overlay these on a single graph (Figure 2). Kmer spectra can provide a number of useful insights. There will be an initial peak around coverage 1, indicating an abundance of kmers with very low coverage, usually due to sequencing errors. Thus the size of this peak can give an indication of sequencing accuracy. There will then be one or more shorter, wider peaks, the number of which can infer the level of heterozygosity in the sample. A single peak indicates a homozygous organism and the peak will occur centered around a point equivalent to the depth of sequencing. The area of this peak can provide an estimate of genome size, useful when sequencing less well-known organisms.

KAT includes several other analyses that are able to assess datasets and assemblies, and the pre-generation of hashes for the readsets paves the way for further analysis.

### 3.2. Mate pair analysis

At TGAC, we have recently started to use the Nextera LMP kits (Illumina FC-132-1001) that can produce libraries with insert sizes of up to 12 kb and are an invaluable tool for scaffolding complex assembles. However, obtaining the best performance from the kits and analyzing them bioinformatically can prove challenging and time consuming. For this reason, we have developed a tool called NextClip (Leggett et al., 2013) which will take sequenced reads from a Nextera LMP library, perform a comprehensive quality analysis and prepare clipped reads suitable for downstream analysis. The tool initially analyses reads for the presence of junction adaptors and classifies reads according to whether the adaptor is present in one, both or neither read. The presence of the adaptor can be an indicator as to whether the read pair is a genuine mate pair or an artefact of the library preparation process. Reads are clipped at the position of the junction adaptor. If a reference is available, NextClip will check the orientation of reads to see if they are in mate pair, paired end or tandem orientation. For each orientation and each category, it will plot insert size histograms. NextClip will also given indications of PCR duplication rates and will generate graphs of GC content versus number of reads, both of which can be important indicators of library quality. Though we continue to work on producing better Nextera LMP libraries, the use of NextClip has enabled us to adjust laboratory procedures in order to produce higher quality libraries with tighter distributions and larger numbers of true mate pairs.

### 3.3. RAD-Seq

RAD-Seq is a method for sampling from the same reduced and representational fraction of the genomes of multiple individuals of a population (Miller et al., 2007; Baird et al., 2008). Typically RAD-Seq uses an inline index within read 1, which connects directly to genomic sequence, as this approach allows early pooling libraries and lower library cost. CASAVA is unable to demultiplex RAD-Seq reads with inline indexes and additional software is required, and as such, two tools are available to perform this—Stacks (Catchen et al., 2011) and RADtools (Baxter et al., 2011).

At TGAC, we have developed a modified version of the RAD-Seq protocol that offsets the position of the restriction enzyme overhang (M. Clark pers. comm.) in a fashion similar to that employed by Genotyping by Sequencing methods such as Elshire et al. (2011). This change in approach ensures that the instrument does not detect an over-abundance of particular bases toward the start of a read, and leads to higher quality base calls. In order to support the offset tags, we developed our own tool, RADplex (Leggett, 2013), which demultiplexes an Illumina read set into separate FASTQ files which can undergo our standard QC analysis with FastQC, Kontaminant and StatsDB.

## 4. Conclusions

In this review, we have described the PAP developed and used at TGAC. As stated in the introduction, we believe that one of the most important aspects of achieving accurate, reliable and informative analysis is to *know your data*. For this reason, along with the use of tools such as FastQC, we are committed to develop our own open-source software for *data-driven informatics*. Tools such as StatsDB, NextClip and KAT are helping us to better understand our sequencing platforms and to tailor lab procedures and downstream analyses to make the most effective use of the data generated.

In the future, we plan to move from the current approach of a single PAP with a number of optional components to one where MISO can automatically choose which components to run based on the nature of the sequencing project. This will constitute automated initiation of the supplementary pipelines described above, as well as pipelines such as RAMPART (Mapleson, 2013) for *de novo* assembly, or the Tuxedo pipeline (Trapnell et al., 2012) for RNA-Seq analysis.

The TGAC PAP is mature for the Illumina platform, and is being expanded to support our other platforms. Inevitably, evolution of the pipeline for all platforms will invariably continue as long as new protocols and technologies become available. We are keen to make use of the wealth of high quality free bioinformatics tools that have been made available to the community and that have been developed as a result of many years of experience and in-depth expertise. However, where there are gaps in the toolset, we will continue to develop our own software and we hope that by making these freely available we will, in turn, be able to support the wider bioinformatics community.

### Conflict of interest statement

The authors declare that the research was conducted in the absence of any commercial or financial relationships that could be construed as a potential conflict of interest.

## Availability

The tools developed at TGAC, along with sample datasets, are available from TGAC's tools and resources page: http://www.tgac.ac.uk/tools-resources/
